# American Academy of Dental Science

**Published:** 1896-06

**Authors:** 

**Affiliations:** American Academy Dental Science


### Reports of Society Meetings.

### AMERICAN ACADEMY OF DENTAL SCIENCE.

THE regular monthly meeting of the American Academy of Dental Science was held at the Parker House, Boston, Wednesday January 1, 1896. Vice-President Dr. William P. Cooke in the chair. The paper for the evening was read by Myles Standish, M.D., Boston. Subject, "Light."

(For Dr. Standish's paper, see page 345.)

DISCUSSION.

*Dr. Hamilton.*—The only practical point that has not been touched upon which comes to me is the influence of the mouth-mirror. Dr. Standish may not know how much we have to use it. We work a great deal by reflected light,—that is, we look at the image of the cavity in a mirror. Now, we must see all parts of the cavity, and this we cannot do without the reflection, so we cannot possibly do without the mirror; but I would like to know if its constant use would have any effect on the eyes.

*Dr. Standish.*—I have never seen a cavity reflected by the mirror, but it brings up a point that I intended to speak of, and that is, the difference between daylight as it comes to us direct and by reflected light. A room that is very well illuminated by light reflected from the wall of a building is a poor light to work in. Schools are usually so arranged that the pupils get the best light, and the teacher generally has to face the windows, through which comes a flood of reflected light. Most of the schools are built in such a way that there is a narrow yard between the school and the next building, and they paint the wall white in order to get all the light possible. Now, that is a very different light from the light that comes to us direct, and it is a very poor light for anybody to work in. It is called a polarized light, meaning thereby that the rays are disarranged from their normal order.

About your mirror, do you have any shadows in your cavity,—that is, would the light reflected from your mirror be sufficient to destroy the shadow?

*Dr. Hamilton.*—I cannot say that I ever noticed that; I should say not.

*Dr. Standish.*—You are obliged to have a certain amount of shadow in order to get your depth, and to know exactly where you should place your instrument without being obliged to look too closely. If the illumination of the cavity by the mirror is sufficient to exactly counterbalance the light from the outside, then we have the difficulty that I spoke of in regard to working in a cross light.

*Dr. Hamilton.*—I hardly think that it can be called a cross light; if it is, then we have to learn to get used to it, as we are obliged to use the mirror almost constantly.

*Dr. Bradley.*—May I ask Dr. Standish if he would consider it working in a cross light, where your chair is in a bay-window, with a window on each side and the chair in the centre.

*Dr. Standish.*—I believe that is the reason why I am here to-night; I think Dr. Hamilton can tell you about that.

*Dr. Hamilton.*—I had a good deal of trouble with my eyes, and as my chair is in a bay-window, after trying a good many experiments, I found that it is best to work with your light coming from one window at a time, and I now have my shades pulled down and let up according as I wish to have light come from the left or the right side.

*Dr. Standish.*—When Dr. Hamilton came to me with a slight inflammation of the eyes, I inquired about the way he received his light, and when he said he worked in a bay-window, I told him that was the trouble, I had no doubt. Soon afterwards I met him and he said that the trouble had disappeared, and that he was taking more notice of where his light came from.

*Dr. Williams.*—That matter of bay-windows I tried myself several years ago, and after a short time I found that I could use but one sash; if I attempted to get the whole flood of light, I found that I got too much cross light, which I soon learned was very trying to the eyes, and so I decided that it was better to have a moderate light come from one source than to attempt to get a great deal of light from two or three sources, and I therefore shut off the light from one side when I wished the light to fall on my patient from another side.

I think Dr. Standish need not apprehend that he would be called "cranky" for his views in regard to the electric lights on our streets. I will read you a part of an editorial, which I clipped from the Boston *Transcript* the other day, which interested me so much at the time that I cut it out:

> "An English paper states that London oculists are up in arms against the very serious danger to the community caused by the electric light. Several eminent eye doctors are agreed on the point that unless a stop is put to the exposure of uncovered electric lights in the streets and in shops and offices, nearly all the population will become blind. Experts are so greatly exercised in the matter that they even suggest that Parliament should take it up, and prohibit the use of plain glass globes for electric lights, unless they be properly shaded."

Just what Dr. Standish has told us to-night, so he need have no fear that he will be looked upon as a "crank" for taking the position which he has in this matter in regard to these intense electric lights in the streets and stores. I have made it a subject for observation for some years, and, I think, we cannot be too careful, not only with regard to the kind of light, but the intensity of the light that we use for our work. If we look at nature, we find that she gives us the sunlight, which has a yellowish tinge, and under ordinary circumstances we can use our eyes all day without tiring them, but in the arc light we have an extreme whiteness which is more like lightning. And, again, comparing with nature we find that lightning comes to us only in flashes and ordinarily from overhead. I had a conversation with one of the agents of the electric light companies when the light first came into general use, and he said that the competition was to get the most light possible. I replied, "You get too much light; such intensity must certainly be ruinous; our eyes are not fitted for such a degree of intensity, and the effects will soon show themselves." We also get too much strength in the incandescent electric light; that needs to be modified, shaded, or diffused in order to enable us to bear it. The Wellsbach gas light has more resemblance to the arc light in its blue or whiteness and is open to the same objection, but its steadiness is one thing in its favor. The new acetylene gas light I took a slight observation of the other day, and that seems to be more intense and whiter than anything yet in common use. I told one of the men there that that was not the proper ambition; what was wanted for the best welfare of the people was to get a strong, good light; one of such strength and color as to be non-irritating to the eye; that we had enough volume of light in its present condition. It struck me, unless they made some provision that the light should always be shaded and modified, there would be great detriment done to people's eyes. There is one club that I know of in Boston, where the reading room is lighted in a most injurious manner. Incandescent electric lights are strung around the room on a level with your eyes, and as you pick up a magazine to read, the light shines directly into your eyes and you soon find it uncomfortable. I went into the side entrance of one of our large variety stores the other day, and at the left, as I entered, there sat a man engraving glass, tumblers, etc. I looked in and saw that he had an incandescent bulb direct in front of his eyes. His eyes were perfectly red, as if he were a bloated drunkard. I stepped up to him and said, "My friend, you are ruining your eyes; you dull the optic nerve by having that light shine into your eyes" (another way of stating that he was using up the purple element of the retina), "and the only way for you to go on safely, if you want to save your eyes, is to put an opaque shade over it, and place it in such a position as to keep the light from falling on your eyes." He thanked me and seemed to appreciate the idea. I don't know whether he has profited by it or not. I have for years made it a practice in reading, etc., to have the light on my left side, and a little to the rear, and I also have it so arranged in my laboratory.

I think we are very much indebted to Dr. Standish for his very clear and practical and scientific illustration of this subject.

*Dr. Smith.*—I would like to ask Dr. Standish, How may we best treat the walls of our operating-rooms in order to avoid the possibility of any irritation which might result from light reflected from them? And, also, if an electric light of say thirty or fifty candle-power, properly shaded and placed over a roll top desk, would be injurious?

*Dr. Standish.*—Both of those questions recall points which I should have spoken of. The first people I saw who suffered from electric lights were book-keepers. With the usual desire for plenty of light and the usual disregard of where it comes from, they took one of these handy incandescent electric lights and hung it down on cords right before them, plumb over their books. The result was, as I have explained, a brilliantly illuminated surface in a dark room, and they suffered a great deal. It seems to me that almost anybody ought to know enough not to look an electric light in the face, and yet that is just about what a great many book-keepers are doing daily. You know the way most offices are arranged, the proprietor, who doesn't do anything with his eyes, usually has his desk up near the window; and the book-keeper, who is constantly using his eyes, is put into the darkest corner of the room. The book-keeper takes an incandescent electric light and puts it right down in front of him, and when he has a dark shade around it, he thinks he has the very best arrangement he can possibly get; but you can easily picture to yourselves the result of such an arrangement. The whole of that light is thrown down onto a square of reflecting white paper, and there the book-keeper sits during the whole day, with eyes attuned to darkness by the wall in front of him, looking at a brilliantly-illuminated square which is playing havoc with the retinal purple. He works there about six weeks and then he goes and sees the oculist, and the advice that I give—and I think I give it perhaps fifty times a year to book-keepers—is to have another light, and preferably a bright gas light, placed just above and behind their heads in such a way as to brilliantly illuminate the wall in front of them. With that modification they go along very well, and I think I can safely say that I know of one hundred book-keepers who have taken off their spectacles and have had no further trouble with their eyes, and all they did was to put this extra light behind their heads.

Another thing: a book-keeper who works continuously in an artificial light should not have white books; the paper should have a slight tinge of yellow, so as not to have a white reflected light from the books. That would be a great advantage if you could only bring people to realize its value.

One of the great paper companies advertises to furnish special papers for books, and I hope they will be more generally used.

With regard to the light of the walls, I would say that I have had a curious experience in this matter. I never used to think anything about walls; there was nothing in the books about walls, and I had never heard them considered in the matter of illumination. But here was another instance where I learned something of value from the ordinary experiences of every-day life.

I had a series of patients, school-children, who came from Gloucester, Rockport, or somewhere out of town, and after six or seven of them had come from the same town, it occurred to me that they came from the same school; and so I made inquiries about the situation of that school. The facts were these: they had built a new school-house and made the windows as big as possible. On one side of the school-house was the ocean, and on the other side was a large pond, or an inlet of the sea, I forget which, so that the light came up reflected from the water on both sides. There was plenty of light without painting the walls at all, so they were left with the white skimming of ordinary plaster. It played so much mischief with the children's eyes that I advised the parents to take their children out of the school. Pretty soon a gentleman came in to see me and said, "I am a school committee-man from (wherever it was). We understand that you are advising the people to take their children out of the school because you claim that the walls are injurious to their eyes. Now, of course, we want to have everything about the school-house all right, and I would like to know what color we shall paint those walls?" Well, he posed me. I didn't know. So I thought a few moments and said, "Well, I'll have to consider that question and let you know later." I looked through the books again; I inquired of all my colleagues, and none of them seemed disposed to help me,—at any rate, I didn't get any practical advice, and so I started in from the scientific end to see if I could find out anything in regard to the irritability of certain colors on the retina. I found that at the red-yellow end of the spectrum the rays were all irritating, while the green and blue were not until you get away out into the violet, which is very dangerous. The deductions to be drawn from those investigations, therefore, were that if you want to paint the walls of a room so as to have the most light and have it agreeable. you must put into a light color a tinge of blue or green. It is not necessary that it should be a pronounced blue or green, but just enough to take off the annoyance from a white wall. The thing was very prettily illustrated in my own case when I built a house at the shore. It was built on a high cliff, about sixty feet above the water, and when the sun is shining the light is strongly reflected from the water into the house. The house was not completed until some time in June, and we moved in without waiting for the walls to be painted, and the result was that we had trouble with our eyes that summer, so that by the next season we had the walls colored and there has been no further annoyance. In deciding on what colors to use, I went to a decorating firm here in the city and said that I wished to talk with some one about the proper colors to use in such a place. A man went down with me to the house to look at it from a decorative point of view. I did not say anything about my notions as to color, as I thought I would wait until I heard what his views were in regard to decoration. After looking over the situation, he said, "Well, we must paint these walls very differently from what we should in the city. In the city, we want to have all the light we can get, and here we have more than we want, and we will have to make a scheme of color very different for this place." And he went to work and laid out a scheme of color, and what pleased me most about it was that every single color that he put onto the walls of that house were purely blue or green, and he arrived at it from a practical, decorative stand-point, probably without knowing the reasons why such colors were the best.

I also had an opportunity for studying this thing when I moved into my new office. I wished to have the rooms as comfortable as I possibly could for the patients, and so I put onto the walls of the waiting-room a very pale, yellow-green. It is just about the color of the newest leaves of the Japanese ivy when they first come out, before they turn a dark green. It is a very light green, and the result is amazing. People come in there with irritable eyes and inflamed eyes and they sit there with their eyes wide open, in a room which has three windows and into which the sun is shining all the time. In my office up-stairs, where I meet the patients for consultation, I wished to have the room colored in such a manner that the light would not affect their eyes, and there I used a dark chocolate brown. That room you cannot illuminate with four gas-burners nearly as much as you can the one below with one,—in other words, those colors absorb the light, and down-stairs there is practically no light lost, and yet my patients are just as comfortable in the waiting-room down-stairs as they are in the office.

Since this matter has been brought so much to my attention, I have had several conversations with educators in regard to school-rooms, and especially with a gentleman by the name of Stone, who is connected with the schools of Cambridge, who has given considerable attention to this subject. And they have a new school-house there which he has decorated, and on his invitation I went over to see it. The walls have less green than mine,—in fact, you would hardly know they were green if you did not look closely; and there is another peculiar feature about that school,—they have no black-boards. They are green-boards. The reason of it is that they noticed that many children complained of their eyes when they came to the black-board work. It seems there is a certain grade in the schools nowadays where the black-board is used a great deal, and they found that many children, who always managed to get along up to that period, would have trouble with their eyes, and they decided that it must be that the contrast was too great between the black-board and the white chalk, and so they had the boards painted green. They are dark, almost black, but still distinctly green. It is an experiment as yet, but so far, at least, it appears to be successful, and I hope it is going to prove the solution of the difficulty which brings so many children yearly to the oculist.

So if you wish to have a room well lighted, you should put into the color of your walls a tinge of blue or green; you can then have them fairly brilliant and still have them comfortable. If you do not want the light reflected at all and yet wish to have a color comfortable to the eye, then you must paint it a brown; but, of course, such a color would not be suitable in a room where you were going to do mechanical work. In my office it would be impossible to do comfortable work a few feet from the window.

*Dr. Banfield.*—I work by a west light, and in the summer-time, when the sun gets round so as to shine directly into my window, the light becomes too strong for either myself or my patient, so that I have had placed outside my window a cream-colored shade, and the light comes through that. And as it comes to me in that way it is bearable and comfortable to work by, and not only is this arrangement desirable for modifying the light, but it also keeps the sun off the window and reduces the heat.

*Dr. Briggs.*—Since Dr. Hamilton's trouble with his eyes, which was supposed to have been caused by working in a bay-window, I have experimented to see if I found any difference in my own case, and I have not been able to find that it made such a difference as has been claimed for it. In the morning with the easterly light I do not get any cross light from my west window, because I am in the way of it, and in the afternoon, with the strong light falling direct on my work, as far as I can see, the light from the east window does not appear to be much of a factor in the matter.

It seems to me this peculiarity about the color of the walls is perhaps more of the key-note in these cases than we have realized, and I was wondering if that was not as much the trouble in Dr. Hamilton's case as the cross light. The walls of his operating-room have a very light yellow tint, which, according to Dr. Standish's remarks about color, is irritating,—my room is covered with a flock paper, of an olive color,—and it occurred to me that with all his shades up and the room a blaze of light, possibly this color might be the cause of his annoyance. I had never had any trouble with my light, but I was anxious to avoid trouble, and as I say, I experimented to see if it made any difference in shutting off my different windows, and I have not found it to be so.

I have very good sight, and the only trouble I have found has been in reading at night by electric light. I have lived in hotels for several years, and in reading there has been this slight annoyance that I speak of, nothing serious, but as my little boy expressed it, "your eyes look as if the sand-man had been round;" there was no marked inflammation, nothing but what would disappear by morning. For the last two months I have been out of a hotel and I have been using gas-light again, and I have had no trouble whatever, so that for ordinary purposes I have found that electric lights are very annoying to my eyes.

*Dr. Robinson.*—I would like to ask Dr. Standish if he considers an oil-lamp a good light to read by if properly shaded.

*Dr. Standish.*—There is only one objection to the oil-lamp that I know of, and that is the heat from it. The eye is always moist from its own secretion, and this heat affects the external layer of the eye by drying up this moisture, and it can be proved by examination to be injurious. But if your light is sufficiently good so that you can sit a reasonable distance from it, I think an oil-light is a very good light indeed.

The thanks of the members unanimously voted to Dr. Standish.

WILLIAM H. POTTER, D.M.D.,
*Editor American Academy Dental Science.*

